# Biobutanol from cheese whey

**DOI:** 10.1186/s12934-015-0200-1

**Published:** 2015-03-05

**Authors:** Manuel Becerra, María Esperanza Cerdán, María Isabel González-Siso

**Affiliations:** Grupo EXPRELA, Centro de Investigacións Científicas Avanzadas (CICA), Departamento de Bioloxía Celular e Molecular, Facultade de Ciencias, Universidade da Coruña, Campus de A Coruña, 15071 A Coruña, Spain

**Keywords:** Biobutanol, Cheese whey, Metabolic engineering, *Clostridium*, Yeast

## Abstract

At present, due to environmental and economic concerns, it is urgent to evolve efficient, clean and secure systems for the production of advanced biofuels from sustainable cheap sources. Biobutanol has proved better characteristics than the more widely used bioethanol, however the main disadvantage of biobutanol is that it is produced in low yield and titer by ABE (acetone-butanol-ethanol) fermentation, this process being not competitive from the economic point of view. In this review we summarize the natural metabolic pathways for biobutanol production by Clostridia and yeasts, together with the metabolic engineering efforts performed up to date with the aim of either enhancing the yield of the natural producer Clostridia or transferring the butanol production ability to other hosts with better attributes for industrial use and facilities for genetic manipulation. Molasses and starch-based feedstocks are main sources for biobutanol production at industrial scale hitherto. We also review herewith (and for the first time up to our knowledge) the research performed for the use of whey, the subproduct of cheese making, as another sustainable source for biobutanol production. This represents a promising alternative that still needs further research. The use of an abundant waste material like cheese whey, that would otherwise be considered an environmental pollutant, for biobutanol production, makes economy of the process more profitable.

## Introduction

### Biobutanol as energy source

The limited availability and rising price of fossil fuels, together with the growing concern on the greenhouse effect and the climate change, urge to evolve efficient, clean and secure systems for the production of advanced biofuels from sustainable cheap sources.

Nowadays bioethanol is the most widely used, blended with gasoline at various percentages, but biobutanol has proved better characteristics. The term biobutanol includes four butanol isomers: *n*-, *sec*-, *tert*- and, mainly, isobutanol with a low melting temperature and the highest octane number (a measure of anti-knock properties). Isobutanol and *n*-butanol are the most largely manufactured. Biobutanol energy value is similar to gasoline and 30% higher than ethanol; it can be used mixed with gasoline up to any level and even pure, while ethanol only can be used mixed with gasoline up to a limited level; since it is not hygroscopic, it can be blended with gasoline at the refinery, while ethanol and gasoline must be blended just before use; also it can be blended up to 40% with diesel or biodiesel and can be upgraded to aviation jet biofuel; biobutanol can be used without modification of existing automotive engines both in pure or blended forms; it is non-corrosive and then also the existing infrastructure of pipelines, tanks, pumps, etc. can be used; and finally its vapour pressure is lower than the one of ethanol, being therefore of safer handling [1-4].

Historically, the ABE (acetone–butanol–ethanol) fermentation process by solventogenic *Clostridium* species was industrially employed from the early 20^th^ century till the Second World War when it was replaced by the production from petroleum. The onset of the renaissance of fermentative production is dated about the 1980s, but up to the 21^st^ century butanol was used just as a bulk chemical; in 2005 the successful use of butanol in an unmodified car completely replacing gasoline was reported, and thereafter interest in biobutanol production for fuel use has been increasingly emphasized [5]. As an example, the web page http://www.biofuelstp.eu/butanol.html (date of reference 08/01/2015) mentions that in December 2013, the company Gevo announced successful trials by the US Army of a 50/50 blend of a Gevo’s fuel in a helicopter.

At present the main disadvantage of biobutanol is that its production through ABE fermentation is not cost-effective compared with other biofuels such as ethanol, since yield and titer of butanol are lower; therefore the improvement of substrates, microbial strains and processes for its cost-competitive production is a matter of priority research [4].

An approach to compare the range of yield and economics of biobutanol and bioethanol productions was made by Pfromm et al. in 2010 [6]. The authors compared the fermentative production of *n*-butanol vs. ethanol from corn or switchgrass in terms of the lower heating value (LHV) of the liquid fuel products per unit mass of the feedstock, and they found that the energy yield of butanol using ABE technology was about half of ethanol with both substrates. Reasons include that the ABE fermentation converts a substantial amount of carbon to acetone which cannot be used as a fuel, that ABE fermentation produces relatively more CO_2_ than yeast alcoholic fermentation, and that more starch remains unfermented. A given fermenter volume produced about one-quarter of the LHV as *n*-butanol per unit time compared to ethanol. They also report, in terms of carbon mass balance, industrially confirmed yields for fuel ethanol production by yeast equivalent to about 0.30 kg pure ethanol per kg corn and, for ABE fermentation by *C. acetobutylicum*, equivalent to about 0.11 kg *n*-butanol per kg of corn (3 kg of starch converted to 1 kg of mixed solvents with a weight ratio of *n*-butanol/acetone/ethanol of 6/3/1). Ethanol fermentation reaches about 15% (equivalent to ~7% of ethanol saturation in water) while ABE fermentation reaches about 2% *n*-butanol (equivalent to ~25% of *n*-butanol saturation in water). To equal the yeast based bio-ethanol process these authors estimate that *n*-butanol yield should increase from about 0.11 to 0.19 kg *n*-butanol per kg corn. Even though during the last years diverse efforts to improve biobutanol fermentative production have been performed, as here reviewed, yield is still a bottle-neck [4] as long as feedstock costs can be the highest fraction of the overall production cost of bio-based liquid fuels; thus on a conventional plant, corn starch is reported to account for up to 79% of the overall solvent production cost [7].

Molasses and starch are main sources for butanol manufacture hitherto. However, molasses show a geographical limitation and starchy raw materials, such as corn and wheat, are food or feed supplies and then are not available for biofuels large-scale manufacture. Moreover, lignocellulosic residues that are plentiful and very cheap have been widely investigated but their recalcitrance to degradation, and the generation of inhibitors of the process, challenge production of butanol. So that alternative sustainable sources for biobutanol production are still needed [4]. A similar situation could be described for bioethanol production. Other waste materials, like cheese whey, that would otherwise be considered environmental pollutants can be converted into energy sources, thus making the pathway to a biofuel-based economy more feasible [8]. Cheese whey is already a substrate for bioethanol production at industrial scale [9] but its use for biobutanol production has been less investigated and needs further research, as here reviewed.

## Review

### The metabolic pathways in the production of biobutanol

#### Natural producers of biobutanol

**Figure 1:**
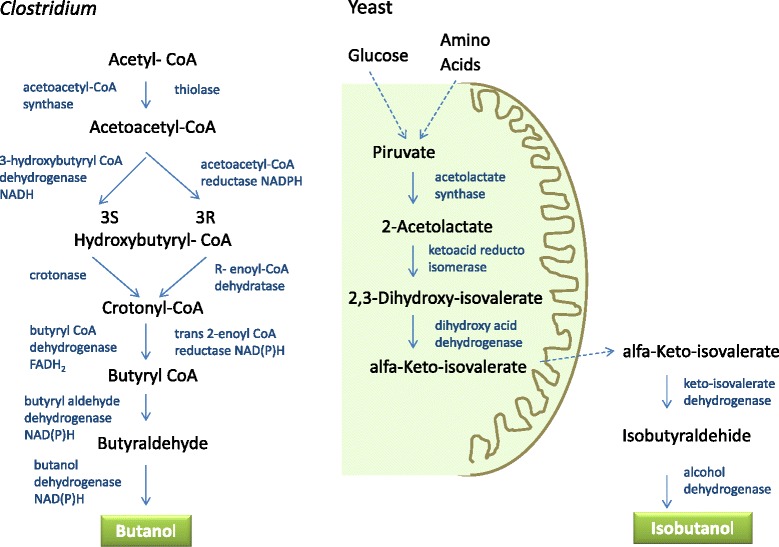
**Scheme of the biobutanol synthesis metabolic pathways in Clostridia and yeast.**

*Clostridium* species are natural producers of *n*-butanol through a biosynthetic pathway that depends on acetyl-CoA sources, the CoA-dependent pathway. Well-known species from Clostridia producing biobutanol are *C. acetobutylicum, C. saccharoperbutylacetonicum, C. beijerinckii, C. saccharoacetobutylicum, C. aurantibutyricum, C. cadaveris, C. sporogenes, C. pasteurianum,* and *C. tetanomorphum*. Among them, the first four species produced the highest yields [10]. In Clostridia acetyl-CoA is produced from diverse carbon sources, also including lactose, the sugar present in whey [11,12]. The fermentation of these sugars usually proceeds in batch mode in two phases. The first (acidogenesis) produces acetic and butyric acids and the second (solventogenesis) acetone, butanol and ethanol (thus named ABE fermentation). In the CoA-dependent pathway two acetyl-CoA molecules are used to generate a C4 molecule, followed by reduction into *n*-butanol. The enzymes, coenzymes and stereo-specificity of each reaction to produce *n*-butanol are shown in Figure 1. The first problem to make this process economically attractive for the production of biobutanol is that it is necessary to find a way to easily shift the metabolism of natural producers from the acidogenic phase towards solventogenesis. Besides, it is necessary to avoid the toxicity of the solvent products, which are mostly attributed to the action of butanol on the microbial cell membranes due to its chaotropic effect [13]. Butanol concentrations over 2% seriously compromise bacterial survival [14].

Another natural metabolic pathway for the production of biobutanol is by deviation of metabolic intermediates from the biosynthesis of aliphatic amino acids in yeast [15,16]. In some yeast species producing fusel alcohols as fermentation by-products, the Ehrlich pathway decarboxylates keto acids and produces aldehydes, which are finally reduced to alcohols. Since keto acids are the precursors of amino-acids, *n*-propanol is obtained from isoleucine, isobutanol from valine and *n*-butanol from norvaline pathways (Figure 1). Isobutanol is the preferred for industrial use because it has a better octane number than *n*-butanol. The industrial application is hampered by the very low intrinsic production in yeast. This route is indeed disadvantageous for the cell since it diverts intermediates, which are necessary for amino acid biosynthesis and therefore it is not naturally favoured.

### Engineered pathways in bacteria

Two main strategies for improving biobutanol production in bacteria can be considered. The first one is based on regulatory and metabolic engineering of bacteria from the Clostridia, and all attempts in this field aim to avoid undesired products and to increase the yield of alcohols obtained as well as the tolerance to butanol. The second one is the engineering of non-producer bacteria with heterologous pathways of biobutanol biosynthesis from natural producers or with artificially generated pathways combining enzymes from different genera. Table 1 summarizes the techniques employed, yield and productivity reached for butanol and ABE.

**Table 1:** **Metabolic engineering approaches to improve biobutanol production by bacteria**

**Bacteria and engineering technique**	**Fuel concentration**	**Productivity**	**Reference**
	**g/L**	**g/L h**	
*Clostridium acetobutylicum* not engineered	B: 8.3	nr	[17]
*Clostridium beijerinckii* engineered to use liquefied corn flour + product recovery	ABE: 81.3	nr	[18]
Hyper butanol producing strain of *Clostridium pasteurianum* isolated by chemical mutagenesis and optimized to produce butanol from glycerol in bach culture	B: 17.8	B: 0.43	[19]
Idem to previous in continuous culture	nr	ABE: 8.30	[19]
		B: 7.80	
*Clostridium ljungdahlii* engineered to use a mixture of hydrogen and carbon monoxide	butanol produced, but only detectable during middle growth phase, then it is metabolized to butyrate		[20]
*Clostridium acetobutylicum* engineered by inactivation of the *adc* gene, encoding the acetoacetate decarboxylase and necessary for acetone synthesis.	B: 14.8	nr	[21]
	BE: 18.1		
*Clostridium acetobutylicum* engineered with a synthetic operon for obtaining isopropanol from acetone	IBE: 35.6	IBE 0.83	[22]
*Clostridium acetobutylicum* engineered for obtaining isopropanol from acetone	IBE: 27.9	nr	[23]
*Clostridium ljungdahlii* transformed with a plasmid, carrying the *C. acetobutylicum* butanol synthesis pathway genes	B: 0.15*		[24]
*Escherichia coli* engineered to produce isopropanol and butanol with genes from *C. acetobutylicum* and others	I: 4.9	I: 0.4 B: nr	[25]
	B: 0.5		
*Escherichia coli* engineered to produce butanol with genes from *C. acetobutylicum*	B: 0.20-0.58	nr	[26]
*Pseudomonas putida* engineered to produce butanol with genes from *C. acetobutylicum*	B: 0.12	nr	[26]
*Bacillus subtilis* engineered to produce butanol with genes from *C. acetobutylicum*	B: 0.02	nr	[26]
*Lactobacillus brevis* engineered to produce butanol with genes from *C. acetobutylicum*	B: 0.30	nr	[17]
Synthetic butanol pathway expressed in *Escherichia coli*	B: 4.65	nr	[27]
Modified *Escherichia coli* by the 2-keto-acid pathway	B: 20	B: 0.18	[25]
Modified *Corynebacterium glutamicum* by the 2-keto-acid pathway	B: 4.9	nr	[28]

*in the middle of the exponential growth phase but consumed by the organism (only 0–15 mg/L left at the end of growth).

A: Acetone; B: 1 or 2-butanol; E: Ethanol; I: Isopropanol; nr: not reported.

### Towards a better solventogenic *Clostridium* producer

Several attempts to improve *C. acetobutylicum* by mutagenesis were successful. A better tolerance to butanol, a higher yield and improved sugar source utilization were achieved by these approaches (reviewed in [5]). Several species of *Clostridium* have been also engineered in order to make possible the use of alternative carbon sources such as: liquefied corn flour [18], glycerol [19] and a mixture of hydrogen and carbon monoxide [20].

A direct way to improve fuel alcohols production is to avoid acetone formation in the ABE process [29,30]. This strategy has been pursued by inactivation of the *adc* gene, encoding the acetoacetate decarboxylase and necessary for acetone synthesis [21]. Another approach consists in obtaining isopropanol from acetone by metabolic engineering. The mixture of isopropanol, butanol, and ethanol (IBE) produced by engineered *C. acetobutylicum* is also useful as biofuel. The gene encoding the required dehydrogenase from *C. beijerinckii* and other genes have been transferred to *C. acetobutylicum* [22,23]. A recently reported IBE strain produces 99% of fuel alcohols with negligible amount of acetone [23]. Usually metabolic engineering is a pyramidal task in which the starting strain has been already obtained by improving metabolic fluxes and yield by a previous approach [23].

### Towards artificially-generated bacterial producers

In the production of butanol by solventogenic Clostridia starchy substrates or molasses are consumed as carbon sources. To avoid competence with nutritional feedstock, an alternative is the utilization of gaseous substrates and acetogenic Clostridia [24]. Following this strategy *C. ljungdahlii* was transformed with a plasmid, carrying the genes *thlA*, *hbd*, *crt*, *bcd*, *adhE*, and *bdhA* of the *C. acetobutylicum* butanol synthesis pathway; these genes encode the thiolase, 3-hydroxybutyryl-CoA dehydrogenase, crotonase, butyryl-CoA dehydrogenase, butanol/butyraldehyde dehydrogenase, and butanol dehydrogenase, respectively [20].

The introduction of the *Clostridium* butanol pathway into other bacteria that grow faster, are more resistant to butanol or metabolize alternative substrates might solve some of the limitations observed in *Clostridium. Escherichia coli* has a higher growth rate than Clostridia and has been engineered to produce butanol [25,31]. *Pseudomonas putida* that overcomes butanol toxicity by specific efflux pumps or *Bacillus subtilis* that becomes more resistant by changing its cell-wall composition are also selected hosts [26]. *Lactobacillus brevis* that has a high tolerance to butanol, and is able to digest C5 and C6 substrates, has also been used [17].

The production of butanol is principally limited by the intrinsic kinetic characteristics and cofactor specificity of the enzymes of the natural *Clostridium* pathway, with independence of the selected host organism. This limitation can be overcome by using a synthetic pathway. This has been done by combining convenient enzymes from four different organisms into a synthetic butanol pathway expressed in *E. coli* [27]. The use of a 2-keto-acid decarboxylase of low substrate specificity together with an alcohol dehydrogenase into a modified *E. coli* strain produces high yields of isobutanol by the 2-keto-acid pathway [25,31,32]. The 2-keto-acid pathway of *Corynebacterium glutamicum* has also been engineered, taking advantage of the high amino-acid production characteristic of this bacteria [28].

In summary, reviewing the main strategies used for biobutanol production in bacteria (Table 1) several conclusions arose. First, using natural producers, the best strategies are those based in selection of strains and fermentation conditions in combination with product recovery techniques that may avoid toxicity [18]; or those that increase fuel concentration by avoiding the production of acetone [21] or deviate this metabolite to the production of isopropanol [22]. These approaches allow a two to ten folds increase of achieved fuel concentration in comparison to the original, not engineered, strains of *Clostridium acetobutylicum*. The introduction of the metabolic pathway for ABE fermentation in other bacteria, considered advantageous because they do not require anaerobic fermentations, grow faster or are more tolerant to butanol, has been less successful since the reached concentration of fuel are far below those obtained for the natural producers [17,25,26]. A promising area is the engineering of non-natural producer bacteria with the 2-keto-acid pathway, which has increased more than two folds the fuel concentration in comparison to natural strains of *Clostridium acetobutylicum* [25].

## Engineered pathways in yeasts

Although in hitherto published papers engineered *Clostridia* or *E. coli* show higher yields of biobutanol production than yeasts [33], it has been reported that industry opts for engineered *Saccharomyces cerevisae* for isobutanol high-scale production systems [2]. One reason may be the good previous operational experience about bioethanol production by this yeast together with the possibility to adapt the same infrastructure. In fact, the companies producing biobutanol by recombinant yeasts are or were bioethanol producers. A visit to the web page http://www.biobutanol.com/ or to those of the companies Gevo, Butamax or Butalco (date of reference 2014-10-06) support these statements. Moreover, there are several drawbacks with the use of bacteria for biobutanol production in industry such as requirement for strictly anaerobic conditions in the *Clostridium* species, a complex separation process from the fermentation media, narrow and neutral pH growth rate and susceptibility to phage infections when grown on a large scale [4,34]. These drawbacks associated to bacterial fermentations support the use of yeast.

*S. cerevisae* has the enzymes to synthesize isobutanol by the two-compartment Erhlich pathway, in which ketoisovalerate (an intermediary product of mitochondrial valine biosynthesis) is catabolized into isobutanol in the cytosol, although levels produced naturally are low (Figure 1). The strategies of yeast metabolic engineering employed by different research groups, from academy or industry, to improve biobutanol production (as reviewed by [2,35]) are varied and can be summarized as follows: Overexpression of *ILV2, ILV5* and *ILV3*, the own genes of the valine biosynthesis pathway encoding acetolactate synthase, ketoacid reductoisomerase and dihydroxyacid dehydratase, respectively; expression of all enzymes involved in isobutanol synthesis into mitochondria using a mitochondrial transport signal; external addition of ketoisovalerate to be converted by heterologous decarboxylases and alcohol dehydrogenases, combined with disruption of the *PDC1* gene, encoding pyruvate decarboxilase; expression of the valine biosynthetic pathway in the cytosol by removing the mitochondrial targeting signals of the genes *ILV2, ILV5* and *ILV3* or introducing heterologous genes, but keeping the mitochondrial pathway intact; replacement of the mitochondrial pathway by a cytosolic pathway to avoid competition, which was got by over-expression of cytosolic forms encoded by *ILV2, ILV5, ILV3, ARO10* or other ketoacid decarboxylase, and *ADH2* or other alcohol dehydrogenase, together with deletion of mitochondrial *ILV2* and the three pyruvate decarboxilase encoding genes (*PDC1, PDC5, PDC6*); increase of cytosolic acetyl-CoA supply (by expression of the *E. coli* pyruvate dehydrogenase complex) together with reduction of pyruvate decarboxilase endogenous activity and expression of the butanol synthetic pathway from Clostridia. Moreover, additional genetic manipulations were performed to overcome the lack of growth on glucose as single carbon source of the pyruvate decarboxilase mutant (pdc-) strains, to reduce other by-products formation besides ethanol, to increase dihydroxyacid dehydratase (encoded by *ILV3* or heterologous genes) activity, or to correct cofactor imbalance. Combining several of these strategies more than 80% of the maximum isobutanol theoretical yield was reached. Also, Brat et al. [36] adapted the codon usage of the cytosolic expressed genes of valine biosynthesis to those of the highly expressed genes of glycolysis.

More recently, during the last 2 years (2013–2014) new yeast metabolic engineering approaches have been published that are following summarized. Also, in Table 2 a selection of engineered *S. cerevisiae* strains, only those producing isobutanol and recently published, is summarized, showing reported yield and/or titer reached.

**Table 2:** **Metabolic engineering approaches to improve isobutanol production by the yeast** ***S. cerevisiae***

**Yeast** ***S. cerevisiae*** **engineering technique and carbon source**	**Isobutanol concentration and/or yield**	**Observations**	**Reference**
Overexpression of biosynthetic genes *ILV2*, *ILV3*, and *ILV5* in valine metabolism. Aerobic batch cultures in 40 g/L glucose.	4.12 mg/g glucose	First report of isobutanol production by yeast. (2011)	[37]
Expression of a cytosolic pathway consisting of *ILV2*, *ILV5*, *ILV3*, *ARO10*, and *ADH2*, and deletion of the first gene of the mitochondrial pathway. Batch cultures in 4% glucose.	630 mg/L,	The highest titer reported up to the date (2012)	[36]
	15 mg/g glucose		
Characterization of an alternative metabolic pathway for butanol and isobutanol production, using glycine as a substrate via glyoxylate and α -ketoacids intermediates.	58 mg/L	Isobutanol from glycine (2013)	[38]
Elimination of competing pathways in strains lacking genes encoding members of the pyruvate dehydrogenase complex (*LPD1*) and resolving the cofactor imbalance by overexpression of enzymes responsible for transhydrogenase-like shunts transforming NADH into NADPH. 24 hours batch fermentation in 100 g/L glucose.	1620 mg/L,	The highest titer reported hitherto (2013)	[39]
	16 mg/g glucose		
Compartmentalization of the Ehrlich pathway into mitochondria.	635 mg/L	Increased isobutanol production by 260% (2013)	[40]
Batch semi-aerobic cultures in 20% glucose.			
Fermentation of D-xylose directly to isobutanol: Overexpression of an optimized, cytosolically localized valine biosynthesis pathway together with xylose isomerase *XylA* from *Clostridium phytofermentans*, transaldolase *Tal1* and xylulokinase *Xks1*, ketoacid decarboxylase *Aro10* and alcohol dehydrogenase *Adh2*.	1.36 mg/L	Isobutanol from xylose (2013)	[41]
	0.16 mg/g D-xylose.		
Genes involved in isobutanol production (*ILV2, ILV3, ILV5, ARO10,* and *ADH2*) overexpressed in an *ald6*Δ*bat1*Δ strain (to eliminate competing pathways) expressing *LEU3*Δ*601* (to activate transcription of endogenous genes in the valine and leucine biosynthetic pathways)	376.9 mg /L	Transcriptional activation (2014)	[42]
Closed tube cultures with 100 g/L of glucose as substrate			

Branduardi et al. [38] proposed an alternative way based in exploiting the catabolic pathway of amino acids resulting from hydrolysis of proteins, where proteins come from dead microbial biomasses at the end of fermentation. The authors demonstrated how glycine could be the substrate for the synthesis of butanol and isobutanol in *S. cerevisiae* following the pathway glyoxylate, β-ethylmalate, α-ketovalerate and α-ketoisovalerate. No heterologous activities were used.

Krivoruchko et al. [34] reconstructed the 1-butanol biosynthetic pathway in *S. cerevisae*. This was done by increasing flux towards cytosolic acetyl-CoA by means of the transformation with a plasmid expressing the genes *ADH2* (alcohol dehydrogenase), *ALD6* (acetaldehyde dehydrogenase), *ACS1/ACS2* (acetyl-CoA synthetase), and *ERG10* (acetyl-CoA acetyltransferase). Also using null mutant strains for the genes *CIT2* (citrate synthase) or *MLS1* (malate synthase).

Si et al. [43] reported the discovery, characterization and engineering of an endogenous 1-butanol pathway in *S. cerevisiae*, dependent on the catabolism of threonine, in a similar way to fusel alcohol production by the Ehrlich pathway. Specifically, the leucine biosynthesis pathway was engaged in the conversion of key 2-keto acid intermediates. Upon introduction of a single gene deletion *adh1*∆ (causing deficiency of alcohol dehydrogenase), overexpression of the Ehrlich pathway enzymes and eradication of the competing routes, the highest reported 1-butanol titer by *S. cerevisiae* was achieved (242.8 mg/L from glucose as substrate).

Matsuda et al. [39] improved isobutanol yield by *S. cerevisiae* strains lacking genes of the pyruvate dehydrogenase complex such as *LPD1*, to reduce competition between the pyruvate supply for isobutanol biosynthesis and acetyl-CoA biosynthesis in mitochondria, together with over-expression of enzymes responsible for transhydrogenase-like shunts, converting NADH to NADPH, such as pyruvate carboxylase, malate dehydrogenase, and malic enzyme, to resolve cofactor imbalance.

Avalos et al. [40] showed that a greater increase in isobutanol synthesis was obtained by compartmentalization of the Ehrlich pathway into mitochondria (about 260%) than by overexpression of the same pathway in the cytoplasm (about 10%), in comparison with a strain overproducing the enzymes that catalyze the first three steps of the anabolic route. The expected benefits of mitochondrial compartmentalization are diverse. First, a local increase in enzyme concentration; second, improved availability of intermediates, avoiding the necessity for exporting them outside mitochondria; and third, it reduces the consumption of intermediates by competing routes.

Brat and Boles [41] described the construction of a recombinant *S. cerevisiae* strain able to produce isobutanol directly from D-xylose. Simultaneous over-expression of a cytosolic route of valine biosynthesis and of the three enzymes xylose isomerase from *Clostridium phytofermentans*, transaldolase and xylulokinase allowed the complementation of the valine auxotrophy of *ilv2,3,5* triple deletion mutants and growth on D-xylose as the sole carbon source. The additional over-expression of *ARO10* (ketoacid decarboxilase) and *ADH2* (alcohol dehydrogenase) conferred the cells the ability to directly ferment D-xylose to isobutanol. This is important because D-xylose can represent over 30% of plant biomass and in this way the economy of lignocelulosic hydrolysates fermentation processes is improved [44].

Park et al. [42] engineered a leucine auxotrophic strain for the production of isobutanol and 3-methyl-1-butanol from the catabolism of valine and leucine. The authors deleted two genes (*ALD6* coding for aldehyde dehydrogenase and *BAT1* of valine synthesis), and increased transcription of endogenous genes in the biosynthetic routes of these aminoacids, by expressing a constitutively active form of the Leu3 transcriptional activator.

Also, genome-scale and proteomic analysis, and physiological adaptations, have been performed to improve butanol and stress tolerance by yeast [45-47].

An overview of the above cited academic papers describing engineered yeast strains to produce isobutanol (Table 2), comparing the yields and titers obtained with such strains, points out to the strain developed by Matsuda et al. [39] as the highest producer showing a titer of 1620 mg/L and a yield of 16 mg/g glucose after 24 hours microaerobic batch fermentation in 100 g/L glucose. Although this comparison is difficult to perform since operation time, media and conditions of culture are different in each case, there is a higher difference with other strains in titer (more than double) than in yield since, for example, Brat et al. [36] report a yield of 14.2 mg/g glucose although with a titer of 630 mg/L from aerobic batch cultures in 40 g/L glucose. During the preparation of this review, Generoso et al. [48] reported a comparison among representative academic yeast strains engineered to produce butanol isomers (1-, 2- and iso-butanol) that supports the same here exposed conclusion.

### Cheese whey as substrate for butanol production

The remaining liquid after extraction of milk casein during cheese manufacturing, cheese whey, is relevant in the dairy industry due to its nutritional composition, since it retains about 55% of milk nutrients, and is used as additive in food industries. But, the amount produced, about 90-95% of the milk volume, generates disposal problems, which requires viable solutions [49]. Over 160 million tonnes per year are estimated to be produced in the world, approximately 9-fold the total cheese production, and the tendency is to increase 1-2% annually [9]. The biochemical oxygen demand (BOD) and the chemical oxygen demand (COD) of cheese whey are 35,000 ppm and a 68,000 ppm respectively [50]. Only a half of total world cheese whey production is transformed into various food products [51] and therefore other uses are necessary to cope out with the problem.

Whey valorisation is generally initiated by diafiltration or ultrafiltration to recover whey protein concentrates (WPC), which have many applications in the food industry [9,52]. During these processes large volumes of a lactose-rich stream are also generated. Indeed, lactose, being 70% of total whey solids, contributes largely to the whey polluting load [9]. The utilization of lactose present in whey or permeates is possible through fermentation to ethanol or butanol [9,51].

### Lactose metabolism in *Clostridium*

**Figure 2:**
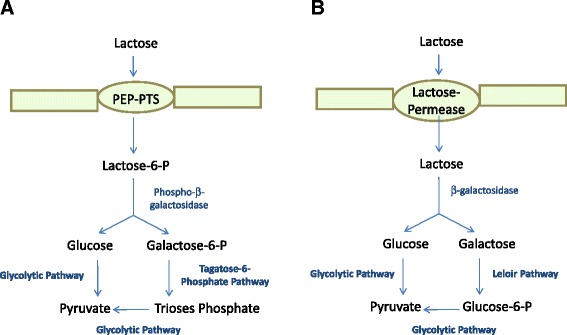
**Schematic representation of the different lactose transport and utilization by** ***Clostridium*** **species. A)** Phosphoenol-pyruvate (PEP)-dependent phosphotransferase system (PTS) pathway and **B)** Lactose permease pathway.

Several strains of *Clostridium acetobutylicum* and *Clostridium beijerinckii* have been used to produce butanol by fermentation of whey. However, most of these studies (88.88% of the works analyzed in this review) were focused on strains of *C. acetobutylicum*. Although relatively little is currently known about the bioconversion of lactose into butanol, several works about molecular characterization of lactose transport and metabolism in *C. acetobutylicum* ATCC 824 strain (the *C. acetobutylicum* most employed strain to ferment the whey, 50% of the studies reported here used this strain) have been published recently [53,54]. In this strain, lactose is taken up via the phosphoenol-pyruvate (PEP)-dependent phosphotransferase system (PTS), which catalyzes the concomitant uptake and phosphorylation and deposits the resulting phosphorylated derivative, lactose 6-phosphate, in the cytoplasm. Lactose 6-phosphate is hydrolyzed to glucose and galactose-6-phosphate by a phospho-β-galactosidase (Figure 2). Glucose may be phosphorylated and metabolized to pyruvate via the normal glycolytic pathway and the galactose-6-phosphate is generally metabolized to triose phosphates by the tagatose-6-phosphate pathway [53]. Both PTS and phospho-β-galactosidase are only induced during growth in lactose, but are absent in glucose-grown cells. Moreover, this strain exhibited a classical diauxic growth, in glucose and lactose medium the *lac* operon is not expressed until glucose is consumed. Therefore, lactose transport and metabolism in this strain seemed to be under the control of a catabolite responsive element characteristic of low-GC gram-positive bacteria [53].

Other *C. acetobutylicum* strains, different from *C. acetobutylicum* ATCC 824, such as *C. acetobutylicum* P262 (the second *C. acetobutylicum* most employed strain to ferment the whey, 43.75% of the studies reported here used this strain), NCIB 2951, NRRL 594, NRRL 598 and NRRL 2490, were shown to contain β-galactosidase besides of phospho-β-galactosidase activities when grown in lactose medium [55], and a β-galactosidase gene has been cloned from strain NCIB 2951 [56]. These Clostridia probably possess a non-PTS mediated transport mechanism and lactose is transported through the cell membrane by a lactose permease (Figure 2), together with PTS uptake [57]. The galactose released from the hydrolysis of lactose by the β-galactosidase is metabolized by the Leloir pathway [54,57]. Interestingly, during the fermentation of whey permeate by these strains, the induction of phospho-β-galactosidase was associated with the acidogenic phase, whereas the β-galactosidase was induced in the solvent production phase [55].

Recently, genomic information on *C. beijerinckii* NCIMB 8052, the other main species used in the fermentation of whey (11.11% of the works analyzed in this review), has been published [58]. This strain shows the presence of 47 PTS genes apparently involved in the metabolism of complex carbohydrates, including multiple genes whose protein products are putatively involved in lactose transport and metabolism [58]. Therefore, it has been proposed that lactose metabolism in *C. beijerinckii* NCIMB 8052 could be similar to *C. acetobutylicum* ATCC 824 [59].

All these studies about the genetics and physiology of lactose uptake and metabolism in *Clostridium* species could have theoretical and practical importance to improve the butanol production from milk whey based on manipulation of these strains and the optimizations of fermentation conditions.

### Lactose utilization by *Saccharomyces cerevisae*

Although *S. cerevisiae* is usually the first choice for industrial processes, it is unable to metabolizee lactose. Nevertheless, S. *cerevisiae* can uptake galactose by a permease coded by the *GAL2* gene. Once, inside the cell, galactose can be further metabolized by the Leloir pathway. Thus, the first attempts to ferment cheese whey by *S. cerevisiae* were based on the pre-hydrolysis of lactose with β-galactosidase [60] or the use of a biocatalyst consisting of yeast co-immobilized with β-galactosidase [61,62], but these processes have limited applicability due to the diauxic growth showed by *S. cerevisiae* in glucose and galactose media. To circumvent this problem, 2-deoxyglucose was used as a selection agent to isolate catabolite repression-resistant mutants of *S. cerevisiae* [63], which were able of using glucose and galactose simultaneously.

Genetic engineering techniques allowed the heterologous expression in *S. cerevisiae* of the β-galactosidase and lactose permease genes from *Kluyveromyces lactis*, performing an intra-cellular hydrolysis of the lactose. Earlier developed recombinant strains were genetically unstable and grew slowly in lactose [64]. Both characteristics have been improved in the later developed strains [65-67].

Other approximation to create lactose-consuming *S. cerevisiae* (Lac+) strains consisted in the production by protoplast fusion of hybrids strains of *S. cerevisiae* and *Kluyveromyces* spp. [68]. The construction of recombinant *S. cerevisiae* strains secreting the extracellular β-galactosidase from *Aspergillus niger* to the medium [69-71] or *S. cerevisiae* expressing and secreting *K. lactis* β-galactosidase [72,73] were also reported. In addition, approximations based on the release of intracellular heterologous β-galactosidase (e.g. from *Escherichia coli* or *K. lactis*) by other means different of secretion have been studied [74,75], also including the use of *S. cerevisiae* osmotic-remedial thermosensitive autolytic mutants [76,77] and cell permeabilization with toluene or ethanol [78].

However, in spite of the interesting work done, most of these studies were focused on the fermentation of cheese whey to ethanol. None of the strategies employed or the recombinant *S. cerevisae* strains generated have been used hitherto to test the butanol production from cheese whey.

### Production of butanol by the fermentation of cheese whey by *Clostridium*

The relatively low sugar content in whey (lactose 40–50 g/L) is generally a disadvantage for its use in fermentation processes and requires prior concentration. However this value is near to optimal for the acetone-butanol-ethanol (ABE) fermentation, in which product inhibition limits the amount of sugar consumed. This, altogether with the ability of *C. acetobutylicum* to ferment lactose directly, reveals whey (or whey permeate) as an attractive alternative substrate for the ABE fermentation. Several authors have outlined the value of cheese-whey for butanol production using various *Clostridium* species and whey types for solvent production in conventional batch fermentation using free cells, and also in continuous fermentation. Although most of the reports were published during the 1980’s and 1990’s, the butanol production from cheese-whey is gaining increasing interest during the last ten years. A brief summary of that is following reported.

### Batch production of butanol from whey permeate

Maddox [79] fermented sulphuric acid whey filtrate using *C. acetobutylicum* NCIB 2951. The whey filtrate contained 53 g/L lactose and, after being supplemented with 5 g/L yeast extract, was adjusted to pH 6.5 and sterilized. A butanol concentration of 15 g/L was obtained after 5 days incubation at 30°C. The ratio of Acetone:Butanol:Ethanol solvents obtained was 1:10:1. If the yeast extract was not added, only 13 g/L butanol was obtained after 7 days incubation.

The effect of whey sterilization by autoclaving prior to batch fermentation using *C. acetobutylicum* ATCC 824 and the effect of agitation were shown to be important variables with respect to the solvent ratio produced and the yield obtained [80]. Generally, agitation was detrimental to solvent production with unsterilized and sterilized whey. Moreover, the correlation between lactose utilization rate and solvent yield was negative [81]. Thus, in order to favor solventogenesis against acidogenesis, it is necessary to select conditions such as low culture pH values or high initial lactose concentrations.

The influence of variations in inoculum pretreatment, medium composition, pH and fermentation temperature on butanol fermentation using whey ultrafiltrate with *C. beyerinckii* LMD 27.6 were also investigated [82]. A decrease in the temperature from 37°C to 30°C greatly raised the concentration of butanol produced and the total amount of lactose fermented from whey ultrafiltrate. Variations of inoculum pretreatment, yeast extract concentration and pH had no effects on butanol production at 37°C. The overall product yields [(kg butanol + kg isopropanol)/(kg carbohydrated utilized)] were comparable between whey ultrafiltrate and glucose medium. But the batch reactor mean productivity (kg/L^.^h), including the lag phase, lowered 2–3 folds whether comparing permeate (lactose) with glucose. The lag phase was longer and substrate conversion rate slower for the permeate fermentations.

Mixtures of glucose and galactose, present in hydrolyzed whey, were also tested in fermentation processes [82]. Although a preference for glucose was observed, glucose/galactose mixtures could be used for butanol production.

One interesting observation in the use of whey as the substrate for fermentation is the unusually high ratio butanol/acetone. Bahl et al*.* [83] reported that the fermentation of whey using various *C. acetobutylicum* strains yielded a butanol:acetone ratio of approximately 100:1. This ratio decreased to 2:1 when synthetic media with only glucose as carbon source was used. The difference in the solvent ratios obtained on these two media, was attributed to the more favourable growth conditions in the whey medium; in particular, the presence of lactic acid and an intrinsically optimum concentration of iron supply that is growth limiting and favors butanol production.

In spite of the work done, in general, whey (or whey permeate) has proved to be a relatively poor substrate when overall reactor productivity in batch fermentation is considered, if compared with starch and molasses substrates [84]. This and the incomplete utilization of lactose, are the two major problems of using whey as a substrate. Since the concentrations of total solvents produced are lower than those produced on conventional substrates, product inhibition does not appear to play a main role in the poor production of acetone and butanol from whey. Most solventogenic strains can tolerate a maximum level of total ABE of 20 g/L [85] and 32.6 g/L [86] for the genetically manipulated hiper-solvent producing strain, *C. beijerinckii* BA101. At these solvent concentrations, the fermentation is completely inhibited. The maximum total ABE reported up to now by strains fermenting whey was 17 g/L [79].

Besides product inhibition, ABE fermentation is affected by other factors such as inhibition by substrate or salt concentration. It is also diminished by dead cells increase, nutrient deficiency, low water activity, accumulation of polysaccharides and other macromolecules, or undesired O_2_ losses in connecting tubes that are produced during the feeding of nutrients into the fermentor [5,87,88]. Remarkably, high concentrations (about 200 g/L) of whey permeate (lactose) did not cause substrate inhibitory effect [87], opposite to glucose that showed inhibitory effects at concentrations greater than 161 g/L [88].

More recently, Foda et al. [89] investigated the suitability of lactose containing substrates in batch fermentation using *C. acetobutylicum* DSM 792 and *C. acetobutylicum* AS 1.224. In aerobic conditions, using lactose medium, *C. acetobutylicum* DSM 792 was better for solvents production than AS 1.224 in laboratory conditions. In bioreactor batch experiments, using lactose and cheese whey media, these authors demonstrated that cheese whey is an excellent substrate for biobutanol production with clear advantages respect to lactose medium (1.5 g/L of butanol production in cheese whey media *versus* 0.71 g/L of butanol in lactose medium).

Another inexpensive non-whey-lactose-based substrate, milk dust powder, for ABE fermentation was evaluated recently [59]. Milk dust is a blend of different milk powders left over after industrial milk packaging with high lactose content. Batch fermentation of milk dust powder by *C. acetobutylicum* and *C. beijerinckii* produced 7.3 and 5.8 g/L of butanol respectively and, similar to previous reports for whey [83], both species favors butanol production over acetone compared to fermentation with glucose.

### Continuous production of butanol from whey permeate

Butanol was produced continuously from whey permeate using cells of *C. beijerinckii* LMD 27.6, immobilized in calcium alginate beads [90]. The best production parameters in these conditions were 30°C during fermentation and a dilution rate of 0.1 h^−1^ or less during the start-up phase. In these conditions the reactor productivities were sixteen times higher than those obtained in batch cultures using free *C. beijerinckii* cells grown on whey media.

Production of solvents from whey permeate was also achieved using adsorbed cells of *C. acetobutylicum* onto bonechar in a packed bed reactor (PBR) in continuous that was operated for 61 days maintaining stable conditions [91]. The maximum solvent productivity (4.1 g/L^.^h) was reached at a dilution rate of 1.0 h^−1^; this value represents a yield of 0.23 g solvent/g lactose utilized. Solventogenesis was favoured at high concentrations of lactose in the whey permeate, while low concentrations stimulated acidogenesis.

Ennis and Maddox [92] investigated the ABE continuous fermentation of whey permeate using the strain *C. acetobutylicum* P262A. Cells were recycled by using a dispositive able of being backflushed and consisting on a tubular cross-flow microfiltration (CFM) membrane plant. Cyclic solventogenic and acidogenic behaviour was observed along the continuous fermentation. However, finally it changed to a predominantly acidogenic state, with only short periods of steady-state solvent production. Solventogenic or acidogenic behaviour is correlated with specific morphological cell forms; thus vegetative cells produce acid, while clostridial cells produce solvent. It has been suggested that is necessary to maintain a balance among the various morphological cell forms (vegetative, clostridial and spores) in order to maintain steady-state solvent production during a long time. In this report [92] the loss of this necessary balance was attributed to morphological changes of *C. acetobutylicum* P262A grown on whey permeate.

Also stable biofilms of *C. acetobutylicum* were prepared for continuous fermentation processes using lactose and yeast extract as substrates; a packed bed reactor, containing hydrophobic plastic carriers was prepared and best results of fermentation were obtained by increasing dilution rate and pH [93].

Yeast extract is a necessary supplement when using whey permeate as a substrate for solvent production, presumably as a nutrient source; effectively Maddox demonstrated that batch fermentation without this supplement performed less well [79]. Raganati et al. [94] investigated the feasibility of butanol production by *C. acetobutylicum* DSM 792 by continuous conversion of un-supplemented deproteinized cheese whey. The experiment was performed for more than 3 months in a PBR using Tygon rings as biofilm carriers. The lowest D (dilution rate) investigated gave best performance for the butanol/solvent production. These results were consistent with those reported by Qureshi and Maddox [91], but Raganati [94] obtained better performances when PBRs were operated in absence of yeast extract and with 28 g/L lactose in the feeding (under close operating conditions).

An assessment of the growth kinetics [11] and of both the growth and the metabolism [95] of acidogenic cells of *C. acetobutylicum* DSM 792 were reported. Both tests were carried out under controlled conditions in a continuous stirred tank reactor using a synthetic medium with lactose as carbon source. The proposed models predict the microorganism growth rate [11] or estimate the mass fractional yield [95] under a broad range of operating conditions, including those necessary for solvents production.

### Fed-batch and batch culture coupled with an in situ recovery process

The economic feasibility of classical fed-batch or continuous cultivation is hampered by solvent toxicity and the biphasic performance of acetone-butanol fermentation, respectively. To avoid these problems, *in situ* recovery processes have been coupled with fed-batch and batch cultures.

Employing silicone membrane and oleyl alcohol as the perstraction solvent, the integrated ABE fermentation by perstraction was investigated using whey permeate and lactose as substrates [96]. Subsequently, 57.8 g of ABE were produced with ABE productivity of 0.24 g/L^.^h. The membrane allowed butanol diffusion into the extractant, but diffusion of acetone and acids were limited, which resulted in a higher ABE yield of 0.37 g/g due to acid re-assimilation.

A batch fermentation process coupled with simultaneous product removal by gas stripping has been assayed [87]. In this way, a semi-synthetic medium containing lactose up to 200 g/L has been completely fermented by *C. acetobutylicum* P262 to 70 g/L of ABE.

An integrated system coupled with liquid-liquid extraction significantly enhanced lactose utilization from whey permeate, using *C. acetobutylicum* P262 immobilized cells in a fluidized bed bioreactor, with feeble reduction in productivity [97].

Later, Qureshi and Maddox [98] coupled ABE production from whey permeate medium supplemented with lactose, using *C. acetobutylicum* P262 in a batch reactor, with ABE removal by perstraction. 136.6 g/L of ABE were produced from 313.3 g/L lactose with a yield of 0.44 g/g and productivity of 0.21 g/L^.^h. Using ABE removal by perstraction, the ratio of acids to solvents was significantly lower than in the control batch process, thus indicating the conversion of acids into solvents.

**Figure 3:**
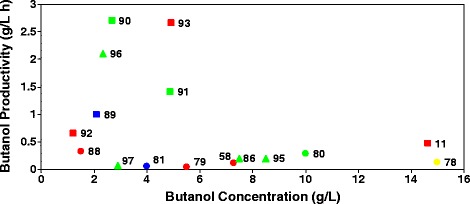
**Butanol productivity (g/L h)** ***versus*** **Butanol concentration (g/L) of** ***Clostridium*** **species growing on lactose medium.** Circle: Batch fermentation; Square: Continuous fermentation; Triangle: Fermentation coupled with an *in situ* recovery process. Green: *C. acetobutylicum* P262 strain; Red: *C. acetobutylicum* ATCC 824 strain; Yellow: *C. acetobutylicum* NCIB 2951 strain; Blue: *C. beijerinckii*. Numbers: references.

The butanol productivity (g/L h) and the butanol concentration (g/L) were chosen to compare the works analyzed in this section (Figure 3). The references [83] and [95] were not included in the Figure 3 because data do not allow comparable calculations. The highest butanol productivity (2.7 g/L h) was reached with *C. acetobutylicum* P262 immobilized by adsorption onto bonechar growing in whey permeate supplemented with yeast extract in a continuous PBR [91], followed (2.66 g/L h) by *C. acetobutylicum* DSM 792 growing in un-supplemented deproteinized cheese whey in a continuous PBR [94]. The highest butanol concentration (15 g/L) was obtained after 5 days incubation by *C. acetobutylicum* NCIB 2951 growing in acid whey filtrate supplemented with yeast extract in batch mode [79] followed (14.64 g/L) by *C. acetobutylicum* DSM 792 growing in synthetic medium supplemented with lactose in continuous stirred tank reactor [11]. In general, batch fermentation gave poor productivity and continuous fermentation processes with *C. acetobutylicum* DSM 792 strain (corresponds to ATCC 824) gave better performance both in butanol productivity and butanol concentration. However, the industrial hurdle rate estimate of 5 g/L h of butanol productivity [99] is far to achieve. Overall, the results support the view that further investigation of whey (permeate) as a substrate for the ABE fermentation is desirable.

### Conclusion and perspectives

The increasing attractive of biobutanol as fuel reveals new opportunities for the utilization of by-products from the dairy industry. The utilization of whey for biobutanol production has been scarcely explored, although this is a desirable objective. Several plants producing bioethanol from whey are operating [9] and the combined production of bioethanol and biobutanol could improve the economic interest and also contribute to obtain a more efficient biofuel. Considering that production of biofuels is a useful mean to slow down carbon dioxide emissions, this is also a green-industry with ecological benefits to humankind and that could contribute to decrease present concerns over global climate change.

The production of biobutanol from whey is theoretically possible since solventogenic Clostridia are able to grow with lactose as carbon source, and direct assays of this production are here reviewed. Proteins, which are as well major components of whey composition, could even be considered as substrates for biobutanol production. Recently an *E. coli* strain was engineered to grow on 13 amino acids as the unique carbon source and it was able to produce 406 mg/L of bio-alcohols (isobutanol, 2-methyl butanol, and 3-methyl butanol) from yeast extract [100].

The establishment of advanced biofuel production from whey sources is still a developing field and a long way remains to be run. Fortunately new tools and strategies are available nowadays to speed the process. Strain development by engineering microorganisms with the appropriate pathways and pathway optimization, eliminating bottlenecks and avoiding toxicity, are now assisted by the “omics” technology and the systems biology approach. New strategies might be used to optimize synthetic combined enzyme activity and the resulting metabolic flux by the “co-localization” concept. The co-localization of diverse proteins participating in the pathway can be achieved in the cell either by enzyme fusion, directed protein trafficking, or the use of scaffolding proteins. All these possibilities have been thoroughly reviewed [101] and could be applied to successful biobutanol production from whey in the near future.
